# An Exquisite Case Report of Follicular Variant of Acinic Cell Carcinoma of the Parotid Gland With Review of Literature

**DOI:** 10.7759/cureus.29559

**Published:** 2022-09-25

**Authors:** Tamanna Adhikary, Harish Kumar, Niva Mahapatra, Abikshyeet Panda, Lipsa Bhuyan

**Affiliations:** 1 Department of Oral Pathology, Kalinga Institute of Dental Sciences, Kalinga Institute of Industrial Technology (KIIT) Deemed to be University, Bhubaneswar, IND

**Keywords:** parotid gland, maxillary, malignant, follicular, acinic cell carcinoma

## Abstract

Acinic cell carcinoma (ACC), previously called acinic cell tumor, is an uncommon malignant neoplasm that tends to recur locally without proper treatment measures. This low-grade neoplasm has four histological subtypes. We hereby report a case of a follicular variant of ACC, which is a rare subtype. A 20-year literature search encompassing this variant of ACC of the parotid region was also performed, which showed four reported cases. A 60-year-old woman reported to our college, Kalinga Institute of Dental Sciences, with the chief complaint of pain and swelling in her upper right back tooth region for the past three months. Upon incisional biopsy, histopathological examination revealed a follicular variant of ACC. Further, immunohistochemistry was also performed using markers such as DOG-1, CK7, S-100, and thyroglobulin, which showed CK7 marker positivity. Subtotal parotidectomy was performed and the tissue was sent for histopathological analysis. Although ACCs are slow-growing and indolent in character, they can frequently recur locally decades later and spread to distant organs. Long-term follow-up is necessary following therapy as ACC has a noticeably high propensity to relapse and create latent metastases.

## Introduction

Acinic cell carcinomas (ACCs) are relatively infrequent low-grade tumors. They comprise 15% of all major salivary gland tumors and 3% of all head and neck tumors [1,2]. They are the third-most common malignant salivary gland, following mucoepidermoid and adenoid cystic carcinomas. Their occurrence is about 80% in the parotid gland, less than 5% in the submandibular gland, and less than 1% in the sublingual and intraoral sites. They constitute about 10% of all malignant tumors in major and minor glands [1]. They are predominantly seen in women and occur in the fifth and sixth decades of life [3].

Earlier they were referred to as acinic cell tumors in WHO 1972 and later when their malignant potential was recognized, they were correctly revised to ACC in the second WHO classification. Histopathologically, four different variants of this entity are seen, namely solid, multicystic, papillary-cystic, and follicular, being the rarest of them [1]. This article focuses on the occurrence of this rare variant in a 60-year-old female.

## Case presentation

**Figure 1:**
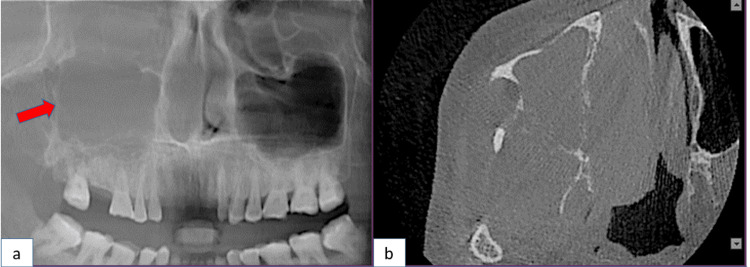
(a) An orthopantomography showing a ground glass appearance, radiopaque in nature. (b) The axial view reveals right maxillary sinus obliteration, along with breach in the posteromedial wall, anterolateral wall, and medial walls of the maxillary sinus.

A 60-year-old female patient was reported to the Oral Medicine Department of the Kalinga Institute of Dental Sciences, Bhubaneswar, with a chief complaint of pain and swelling in the right upper back tooth region for three months. Her previous medical history as well as her family history were irrelevant. She had no risky habits. The right face appeared significantly asymmetrical during an external oral examination. Both the temporomandibular joints and her mouth opening were normal. Past medical history showed sinus surgery done two years back, but the details of the examination were unavailable. On inspection, the swelling firmly adhered to the overlying skin. On palpation, it presented as a firm, immobile, painful slow-growing mass in the right parotid topography, measuring 4 cm in diameter. A bony expansion was present in the palatal region, spanning from the right maxillary first molar to the third molar, and in the maxillary posterior vestibular region. The maxillary sinus obliteration, along with the bony expansion occurred because of the invasion of the tumor entity. Clinical, radiographic, and CT investigations were conducted. The tumor was not visible on CT. The hematological investigations such as bleeding time, clotting time, and hemoglobin count were done and were within the normal limits. Radiographic examination revealed a radiopaque ground-glass appearance in the maxillary posterior region (Figures 1A, 1B).

**Figure 2:**
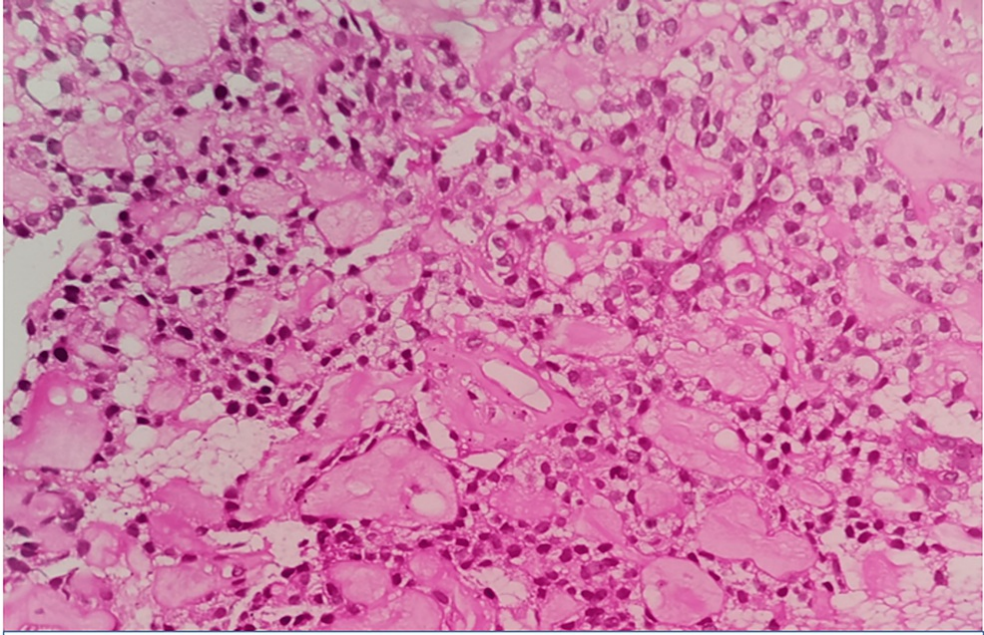
Serous acinar cells with granular basophilic cytoplasm, darkly stained eccentric nuclei (H&E, 400x)

An incisional biopsy was performed and sent for histopathological examination. On gross examination, multiple bits of greyish-white soft tissue were found, the largest measuring 0.8 x 0.5 cm in diameter and the smallest 0.5 x 0.5 cm. On hematoxylin and eosin-stained sections, the lesion was composed of malignant epithelial cells showing features of glandular epithelium arranged in a follicular pattern, as well as a regimented pattern in a few areas. The cells exhibited eosinophilic, granular-to-vacuolated-to-clear cytoplasm with a centrally located vesicular nucleus, and the follicular space was filled with eosinophilic proteinaceous secretory material (Figure 2). This was diagnosed as a follicular variant of ACC. A right subtotal parotidectomy was performed for a duration of three hours. It was discovered during the surgery that the facial nerve branches were not connected to the lesion and that the lesion was limited to the superficial lobe only; therefore, the facial nerve branches were preserved. As of yet, there have been no postoperative complications. The follow-up visits of the patient were eventless.

## Discussion

ACC is considered the least aggressive type of salivary gland tumor. Patients with cases of ACC report to the hospital with parotid or facial region pain in nearly one-third of the cases [2]. ACC is a primary glandular tumor with differentiation toward the terminal intercalated ductal acinar unit and one or more of the following histologic features [4]. Facial nerve paralysis is seen in 3%-8% of patients [5]. In this case, there were no such signs of facial paralysis. Initially, it was given by Nasse in 1892, who referred to it as a blue dot tumor because of the presence of intracytoplasmic zymogen granules. It is the third-most common major salivary gland malignancy. ACC is found in about one out of every six parotid tumors, according to nationwide research in the Netherlands, which found that 15% of parotid tumors were ACC [5].

ACC is also called adenocarcinoma and is composed of cells differentiated toward serous acinar cells. Women (58.8%) are more likely than men to be diagnosed (41.2%) [4,5]. The typical age of diagnosis is 52 years, which is younger than the median age of diagnosis for most other salivary gland cancers. Approximately, 16% of patients were under the age of 30, and women made up a major portion of the population. This age group has a higher proportion of instances (64.4%), compared to those aged 30 and above (64.4%). The parotid gland accounts for 81%-98% of ACCs in the salivary glands, 11% in the submandibular gland, and 3%-12% in the minor salivary glands, the majority of which are in the palate. ACC of the head and neck region has been documented in the small salivary glands, oral cavity, lips, palate, larynx, mandible, nose, and paranasal sinuses, in addition to the major salivary glands. The pancreas, stomach, lung, breast, and prostate are all potential sources of ACC in the human body [6].

Untreated ACCs usually appear as solitary, encapsulated, grey-white soft tumors. The tumor in recurring lesions is frequently lobulated, the capsule may be missing, and necrosis may be visible [6]. Several cell types and growth patterns are noted. The cell types found are acinar cells, vacuolated cells, clear cells and a fourth type designated as non-specific glandular cells.

The acinar cells are relatively large cells that have basophilic to amphophilic cytoplasm with darkly stained nuclei. The vacuolated cells contain clear cytoplasm and vacuoles of varying size and number and are seen in mostly microcystic and papillary-cystic types. The clear cells are similar to acinar cells in size and shape, with the only difference being clear cytoplasm and PAS-negative stain [1].

Histopathologically, four different growth patterns can be found: solid, multicystic, papillary-cystic, and follicular. The follicular pattern is the least common variant of ACC, accounting for 5% of all salivary glands [7]. The solid or classic pattern consists of well-differentiated and uniform basophilic acinar cells, arranged closely in sheets, nodules or an indefinite arrangement. Necrosis is common in such cells with the presence of unusual mitotic figures. The microcystic variant consists of numerous cystic spaces, in which the microcysts may be filled with mucinous or proteinaceous material. The papillary cystic pattern also consists of cystic spaces, but they are greater in size than microcysts are, with the presence of hemorrhage. Nuclei do not have ground glass appearance or grooves. The follicular growth pattern, the least common of all four patterns, resembles thyroid follicles with the septa of epithelial cells surrounding spaces containing a homogenous eosinophilic proteinaceous material. These have cuboidal cells or elongated cells, with calcifications commonly seen [1].

The variable histologic appearance and uncommon occurrence account for its diagnostic dilemmas. The immunohistochemistry for this tumor is positive for CK7, (Anoctamin-1) DOG-1, CK18, Bcl-2, IgA, low molecular weight cytokeratins, EMA, amylase and S-100 in various cases [1,4].

**Figure 3:**
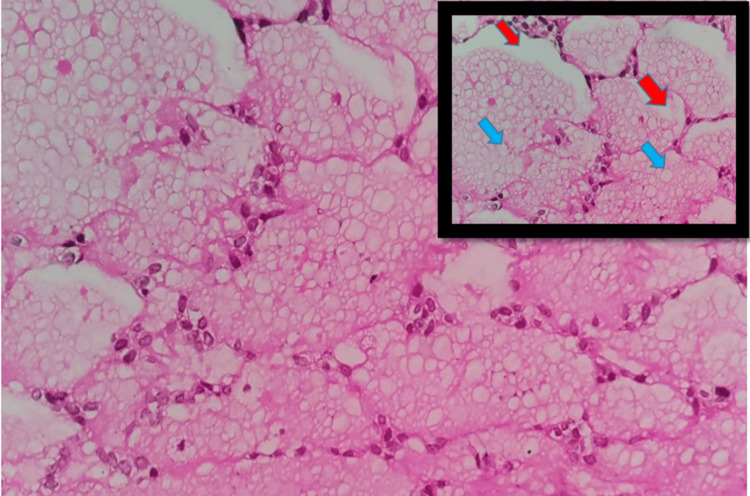
Follicular pattern with central eosinophilic material with presence of clear (blue arrows) to vacuolated (red arrows) cytoplasm (H&E, 400x)

**Figure 4:**
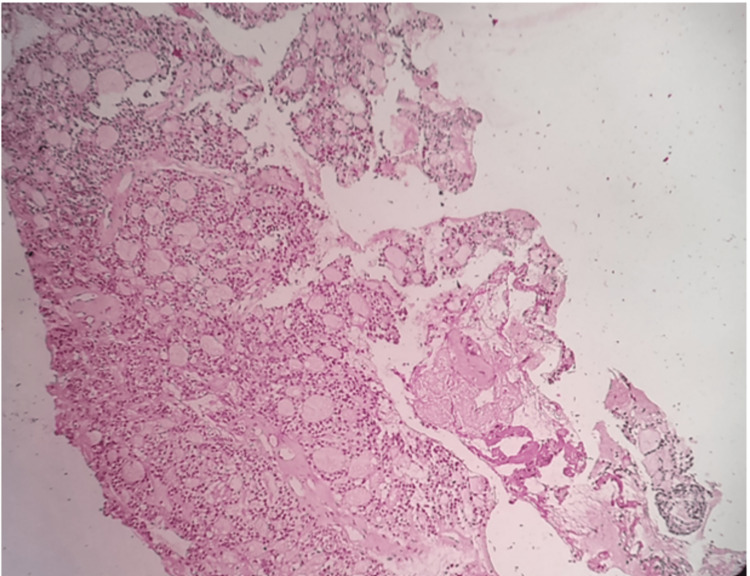
PAS-stained section of serous acinar cells (H&E, 100x)

In this case, the patient had a follicular variant of ACC. This variant is the least frequent and comprises multiple closely packed round cystic spaces filled with homogenous eosinophilic colloid-like material resembling thyroid follicles (Figures 3, 4). We have reviewed the last 20 years of the literature on this follicular variant occurring in the parotid region, as a result of which we found only four reported cases (Table 1).

**Table 1:** Review of literature (2001-2022)

SL NO	AUTHORS	AGE	GENDER	SITE	KEY FEATURES
1	Rimjhim et al.2014 [4]	40	Female	Parotid region	Firm to hard painful swelling, Firmly adherent to the overlying skin, Cells with finely granular basophilic cytoplasm, Solid, acinar and follicular pattern, No lobular architecture, Small microcystic spaces with few large spaces, Focal collection of round cystic spaces filled with homogeneous eosinophilic material lined by duct like cells.
2	Bhagya Lakshmi et al. 2014 [7]	45	Female	Parotid region	Hard, immobile painless swelling, Sheets of cells arranged in follicular pattern with central eosinophilic material resembling thyroid follicles cystic areas and papillary formations with predominant follicular pattern.
3	Yavas et al.2016 [2]	46	Female	Parotid region	Soft, mobile mass which is almost 2cm diameter, Microcystic and follicular structures with luminal secretion, Uniform cells – no atypia or mitosis, Majority of cells - polygonal-shaped, dark basophilic, granular cytoplasm, Others - narrower and cuboidal, amphophilic cytoplasm.
4	Sharma et al.2019 [8]	62	Male	Parotid region	Firm to hard, fixed and non-tender swelling of size 15 x 10 cm, Circumscribed tumor showing features of follicular variant of acinic cell carcinoma with amyloid-like material.

**Figure 5:**
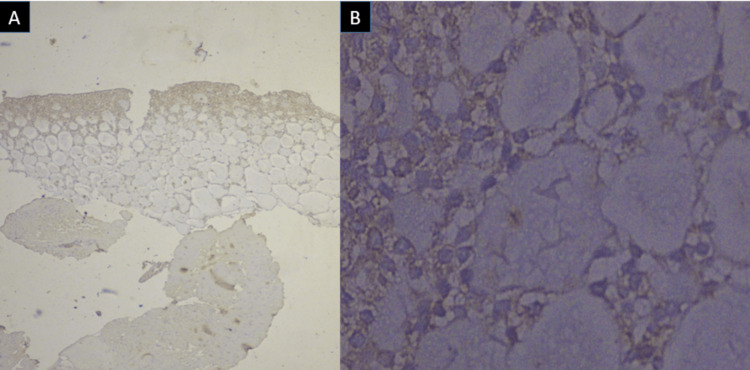
(A) Immunohistochemistry with CK7 positivity x100 magnification, (B) CK7 positivity x400 magnification

The colloid-like material is highly PAS-positive and diastase resistant. The follicles are lined by intercalated duct-like cells and non-specific glandular cells. Thyroglobulin immunostaining is used to differentiate ACC from thyroid neoplasm. We also immunohistochemically analyzed the given section using the markers DOG-1, CK7, S-100 and thyroglobulin. The results showed positivity for the CK7 marker and negativity for the rest of the three markers (Figures 5A, 5B).

The negative staining for thyroglobulin differentiates it from thyroid carcinomas. The recurrence rate averages 10%-35% and distant metastasis rates 13%-16%, in which lungs and bone are the most common sites for distant metastasis. ACC has a good survival rate, with the survival rates ranging from 78%-90%, 63%-83% and 44%-67% at 5, 10 and 20 years, respectively [8].

## Conclusions

ACC is a type of low-grade malignant salivary gland tumor that tends to metastasize and recur locally, the treatment of choice for which is complete excision. The relevance of histology and immunohistochemistry in the therapy of salivary gland malignancies is highlighted in this example, as clinical diagnosis might be deceiving. Hence, histology and immunohistochemistry are important in the early therapy of the patient, improving the prognosis.
